# Challenges associated with the use of ChatGPT in health research

**DOI:** 10.31744/einstein_journal/2026CE1471

**Published:** 2026-01-02

**Authors:** Marcos Henrique de Castro e Souza, Érika Mageste de Almeida Candido, Warley Oliveira Silva, Flávio Narciso Carvalho, Maria Eduarda Finoti Alberice, Antônio Márcio Resende do Carmo

**Affiliations:** 1 Universidade Federal de Juiz de Fora Juiz de Fora MG Brazil Universidade Federal de Juiz de Fora, Juiz de Fora, MG, Brazil.; 2 Faculdade de Ciências Médicas e da Saúde de Juiz de Fora Juiz de Fora MG Brazil Faculdade de Ciências Médicas e da Saúde de Juiz de Fora, Juiz de Fora, MG, Brazil.

Dear Editor,

Vieira et al.^(1)^ report titled "ChatGPT: immutable insertion in health research and researchers’ lives", and Daungsupawong et al.^(2)^ subsequent comments on that report consider the use of artificial intelligence (AI)—specifically ChatGPT—in health research, and discuss the advantages that this technology offers. Daungsupawong et al.^(2)^ complement Vieira et al.^(1)^ report by suggesting the association of new technological resources to expand the application of AI in health research. Both articles warn of potential errors and failures associated with the use of these tools that may arise in the future. However, some important considerations that have been broached should be addressed in greater depth, such as the risk of "hallucinations" and the generation of incorrect responses by AI.^(3)^

The term "hallucination" is used to describe situations in which AI platforms such as ChatGPT create information that was not in the training data, and does not align with reality. The phenomenon is particularly concerning in areas such as healthcare, where the accuracy and reliability of information are crucial. If not properly detected and corrected, these hallucinations can compromise the quality of research, lead to errors in diagnosis and treatment, and hinder decision-making by healthcare professionals.^(4)^

It is essential to acknowledge that AI does not have the capacity to discern the truthfulness of the sources it uses to generate its responses, thus it may incorporate outdated, inaccurate, or false data. This underscores the importance of researchers maintaining a critical view when using these tools, in an effort to ensure that the information generated is rigorously verified before it is applied in scientific or clinical contexts.^(5)^

In conclusion, while AI offers significant potential to transform healthcare research, it is essential that researchers, professionals, and the broader academic community are aware of the present limitations of its use.

## Data Availability

The content is already available.
